# Quality of life and upper limb disability in Charcot-Marie-Tooth disease: A pilot study

**DOI:** 10.3389/fneur.2022.964254

**Published:** 2022-10-05

**Authors:** Laura Mori, Cristina Schenone, Filippo Cotellessa, Marta Ponzano, Alessia Aiello, Maria Lagostina, Sara Massucco, Lucio Marinelli, Marina Grandis, Carlo Trompetto, Angelo Schenone

**Affiliations:** ^1^Department of Neuroscience, Rehabilitation, Ophthalmology, Genetics, Maternal and Child Health, University of Genoa, Genoa, Italy; ^2^Department of Neuroscience, Istituto di Ricovero e Cura a Carattere Scientifico (IRCCS) Ospedale Policlinico San Martino, Genoa, Italy; ^3^Department of Health Sciences (DISSAL), University of Genoa, Genoa, Italy; ^4^UOC Medicina Fisica e Riabilitazione, Istituto Gianna Gaslini, Genoa, Italy

**Keywords:** Charcot-Marie-Tooth disease, hand function, disability, outcome measures, quality of life

## Abstract

Charcot-Marie-Tooth (CMT) patients present mainly lower limbs disability, with slowly progressive distal muscle weakness and atrophy, but hands impairment is a relevant problem affecting the quality of life (QoL). The evaluation of the upper limb is of primary importance. Often these patients present subclinical disorders or report difficulties in manipulating objects, with little evidence in the most used outcome measures. We aim to investigate the impact of hand impairment in the perceived QoL of CMT persons and secondly whether the Disability of Arm, Shoulder and Hand (DASH) scale can be useful in assessing upper limb abilities in CMT. We recruited 23 patients with confirmed genetic diagnosis of CMT. We performed a clinical evaluation with Sollerman Hand Function Test (SHFT), Thumb Opposition Test (TOT) and CMT examination score (CMTES). We completed the clinical assessment with DASH scale and the Short form 36 (SF36) questionnaire for a subjective evaluation of upper limb disability and quality of life. All patients also underwent an instrumental evaluation with a hand-held dynamometer measuring hand grip and tripod pinch and a sensor-engineered glove test (SEGT) to evaluate finger opposition movements in a quantitative spatial-temporal way. As expected, we found significant differences between CMT and control group performances in both clinical and instrumental assessment. Concerning QoL, we found that total score of SF36 and the SF36 Physical Composite Score (PCS) correlate with all clinical and instrumental Outcome Measures (OMs), particularly with Tripod pinch strength and TOT, which are considered major determinants of manual dexterity in CMT. DASH scale correlates with most clinical and instrumental OMs. Not surprisingly, we also found a correlation with DASH work, because CMT affects young patients engaged in work activities. However, we found a low correlation with the TOT and the dynamometer suggesting that DASH may not be the best scale for remote monitoring of upper limb disorders in CMT patients. Nevertheless, the results of our study confirm the usefulness of SF36 in recognizing the impact of upper limb disability in these subjects suggesting its use even in the remote monitoring of physical functioning.

## Introduction

Charcot-Marie-Tooth (CMT) disease is the most common hereditary neuropathy. There is an important variability in the clinical expression of the disease. Symptoms usually start in the 1st−2nd decade of life with slowly progressive distal muscle weakness and atrophy.

The first symptom reported by the patient is usually a difficulty in running, combined with an awkward and uncertain march, with frequent falls. Ankle and toes dorsiflexors are the most affected muscles and patients often complain of joint tightness, deformities, and altered proprioception that further impair muscle function, gait, and balance (1–4).

Upper limbs involvement is usually late and less severe, with deficit of the intrinsic muscles of the hand, loss of opposition, grip strength and dexterity and consequently difficulties in manipulating objects (5). Some subjects also present sensory disturbances, such as paraesthesia, superficial and deep hypoesthesia, hypopallesthesia, usually of a mild entity. Manual function is affected in more than 70% of patients and may require a detailed rehabilitative program with daily exercises (5, 6). It can be present early but is often poorly assessed (7).

Several studies have assessed the relation between strength and dexterity parameters in CMT (8, 9), but not all involved standard or validated tools. Some authors believe that tripod pinch strength and thumb opposition are the main determinants of manual dexterity in CMT1A and should therefore be at the center of intervention strategies that aim to preserve or improve manual dexterity in this disease (9–11).

To study upper limb deficits of CMT patients, specific outcome measures (OMs) are required (12, 13). Unfortunately, the data on upper limbs management and their evaluation are limited, and generally are obtained from small cohorts of individuals. The most used are the Sollerman Hand function Test (SHFT), the Thumb opposition test (TOT), the nine-hole-peg test (9-HPT) and the strength evaluation of tripod pinch and hand grip assessed with an analogical dynamometer. These latter measures are considered major determinants of manual dexterity in CMT (9).

The CMT neuropathy score (CMTNS) and the CMT examination scores (CMTES) include two items focused on the upper limb and have been used in several studies. Between the subjective OM, the most used is the Disability of the Arm, Shoulder, and Hand (DASH) questionnaire.

However, clinical tests are not always sensitive enough to detect the impairment and the modifications over time. An alternative approach to evaluate hand impairment is the sensor-engineered glove test (SEGT), which is a tool already recognized to be useful in measuring hand dexterity quickly, accurately and in a non-invasive way. It is a reliable tool, with good results in the test-retest performed in previous studies (12). The test is rapid, easily repeatable by the patient, requires a little training only and does not suffer from *ceiling effect*, since it reports quantitative measures (13) and, unlike clinical scales, there is no higher limit for the produced values. The SEGT is able to discriminate between normal controls and genetically confirmed patients both with a subclinical impairment of the upper limbs or clinically affected hands. It has been used also to detect the presence of overwork weakness in CMT patients.

The present study aims to investigate the impact of hand impairment, including strength, dexterity, and functioning, in the perceived quality of life of CMT persons. Secondly, we aim to understand whether DASH scale is indicative of upper limb abilities in CMT, by confronting it with both clinical and instrumental OM and therefore may be useful in future studies.

## Materials and methods

We recruited CMT patients with confirmed genetic diagnosis of CMT, attending the multidisciplinary outpatient clinic for the diagnosis and treatment of inherited peripheral neuropathies at the IRCCS Ospedale Policlinico San Martino of Genoa, Italy. Exclusion criteria were: underage subjects, previous hand surgery, other neurological diseases, inflammatory arthritis, diabetes, hearing loss, and uncontrolled pain.

The protocol was approved by the local ethical committee (ClinicalTrials.gov, Identifier: 113REG2017).

### Clinical OMs

All subjects underwent a clinical evaluation by means of SHFT, executed following the published protocol, TOT, as described by Kapandji (14), and CMTES. We also assessed upper limb strength on the following muscles: deltoid, biceps, triceps, wrist and finger extensors, abductor of the thumb, I interosseous.

We completed the clinical assessment with the DASH and Short form 36 (SF36) questionnaire for a subjective evaluation of upper limb disability and quality of life.

The DASH questionnaire consists of 30 questions that inquire about symptoms and functions of the upper limbs (15). These provide a single main score, the DASH function/symptoms (DASH-FS) score, which is basically a summation of the responses. In addition to the 30-item core, there are two optional four-item question sets, the DASH sport/music (DASH-SM) and DASH work (DASH-W), which are scored similarly. The questions test the degree of difficulty in performing a variety of physical activities because of arm, shoulder or hand problems. They also investigate the severity of pain, activity-related pain, tingling, weakness and stiffness and the effect of the upper limb problem on social activities, work, sleep, and self-image. The two optional domains contain activity specific items concerning the performance of sports and/or the playing of musical instruments, and the ability to work (16).

The SF-36 consists of 36 questions on the general health status of patients. This questionnaire provides eight separate scale scores [Physical Functioning (PF), Role-Physical (RP), Pain (P), General Health (GH), Vitality (VT), Social Functioning (SF), Role Emotional (RE), Mental Health (MH)] which are then aggregated into two main scores; the physical composite score (PCS) and the mental composite score (MCS).

### Instrumental OMs

The maximal isometric voluntary contraction of both hands was assessed by a hand-held dynamometer (Citec CT 3001, CIT Technics BV, Groningen, The Netherlands) measuring hand grip and tripod pinch in both. Both measures were performed according to a standardized testing procedure (9, 17, 18).

The SEGT was used according to a previously published protocol. It was used to evaluate finger opposition movements using motor sequences in a quantitative spatial-temporal way in both dominant (DH) and non-dominant hands (NDH). An “eyes-closed paradigm” was chosen to avoid possible confounding effects because of the integration of acoustic and visual information. The patients were instructed to execute finger opposition movements of different complexities: finger tapping (FT) sequence (opposition of thumb to index) and index-medium-ring-little (IMRL) sequence (opposition of thumb to index, medium, ring, and little finger) at maximum velocity. The tasks comprised the execution of a repetition of each sequence, lasting 30 s, with alternate hands. Data were processed with customized software from Glove Analyzer System which permits selection to acquisition and experimental protocol. The following parameters were measured: touch duration (TD) or contact time between thumb and another finger (in ms); inter-tapping interval (ITI) or time between the end of the contact of the thumb and another finger and the beginning of successive contact (in ms); movement rate (MR) [1/(TD+ ITI)] or frequency of complete motor task (in Hz). The MR has been recognized to be the most sensitive glove parameter to detect differences between subjects affected by CMT and controls (12).

Patients with some limitation of the range of motion of their hands or weakness were encouraged to try to complete the task or to make the efforts to perform at their better possibilities. The SEGT software calculated the total amount of touches and recorded any “errors” in the sequence.

### Statistical analysis

Results were reported as mean (SD) for continuous variables and as N (%) for the categorical ones. To evaluate the correlation between variables the Spearman correlation coefficient was calculated. A *p* < 0.05 was considered statistically significant. Differences between HS and CMT performances were analyzed with Mann–Whitney test or *t*-test based on the distribution of the variables. All analyses were performed using Stata version 16.0 (Stata Corporation, College Station, TX, USA) and R v3.5.

## Results

A total of 25 patients were enrolled. One subject was excluded because of pyrexia the day of the assessment, another because the diagnosis of CMT was not genetically confirmed. The mean age of the remained 23 patients was 52.7 ± 13.9, range 28–83 yo and the male/female ratio was 1.6. The CMT subtype distribution of participants in this study was 30% of CMT1A, 48% of CMT1X, 13% CMT1B, 9% CMT2A (Table 1). Only one person was left-handed. At the CMTES score, 7 subjects fell into the category of moderate disability, while the other 16 of mild disability (19). No subjects had severe disability scores.

**Table 1:** Demographic and clinical characteristics of CMT patients.

	**CMT patients**
*N*	23
**Age**	
Mean (SD)	52.74 (13.96)
**Sex**, ***N*** **(%)**	
Male	15 (65%)
Female	8 (35%)
**CMT type**, ***N*** **(%)**	
CMT 1A	7 (30%)
CMT 1B	3 (13%)
CMT 1X	11 (48%)
CMT 2A	2 (9%)
**DH**, ***N*** **(%)**	
Dx	22 (96%)
Sn	1 (4%)
**CMTES**	
Mean (SD)	8 (5.26)
Range	0–20

We also tested a group of 15 age matched healthy subjects with a mean age of 48.5 ± 11.7, range 29–72 yo and male/female ratio of 0.8.

### CMT vs. HS

**Figure 1:**
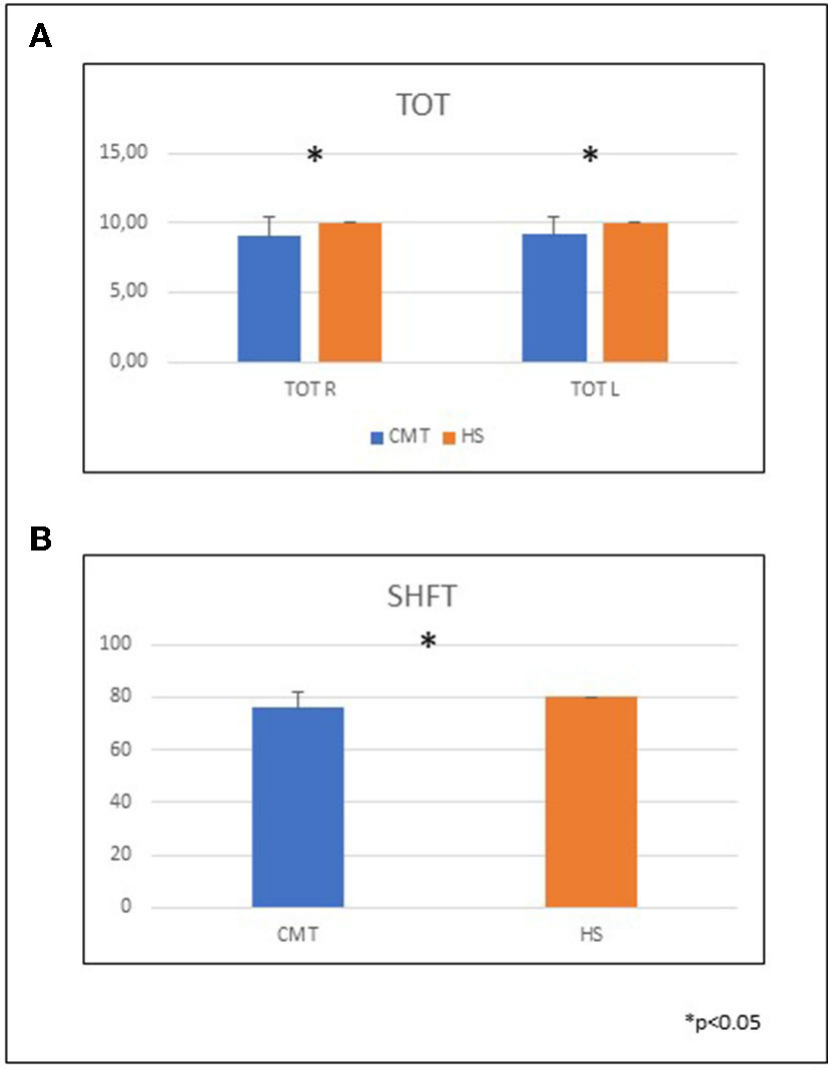
CMT performances in clinical tests compared to healthy subjects. **(A)** Mean and SD of the TOT in the two groups. TOT DH CMT 9.05 ± 1.43; HS 10 ± 0; TOT NDH CMT 9.18 ± 1.18; HS 10 ± 0. **(B)** Mean and SD of the SHFT in the two groups. CMT 75.9 ± 5.9; HS 80 ± 0. TOT, thumb opposition test; SHFT, Sollerman hand function test; CMT, Charcot-Marie-Tooth; HS, healthy subjects; DH, dominant hand; NDH, non dominant hand.

In CMT patients all performances in clinical tests were significantly impaired compared to normal controls (Figure 1) in which we did not find differences between DH and NDH.

CMT subjects presented worse results at the dynamometer in both tripod pinch and hand grip, although not significant. CMT upper limb strength was similar in DH and NDH, while in healthy subjects DH was stronger than NDH (*p* < 0.001).

**Figure 2:**
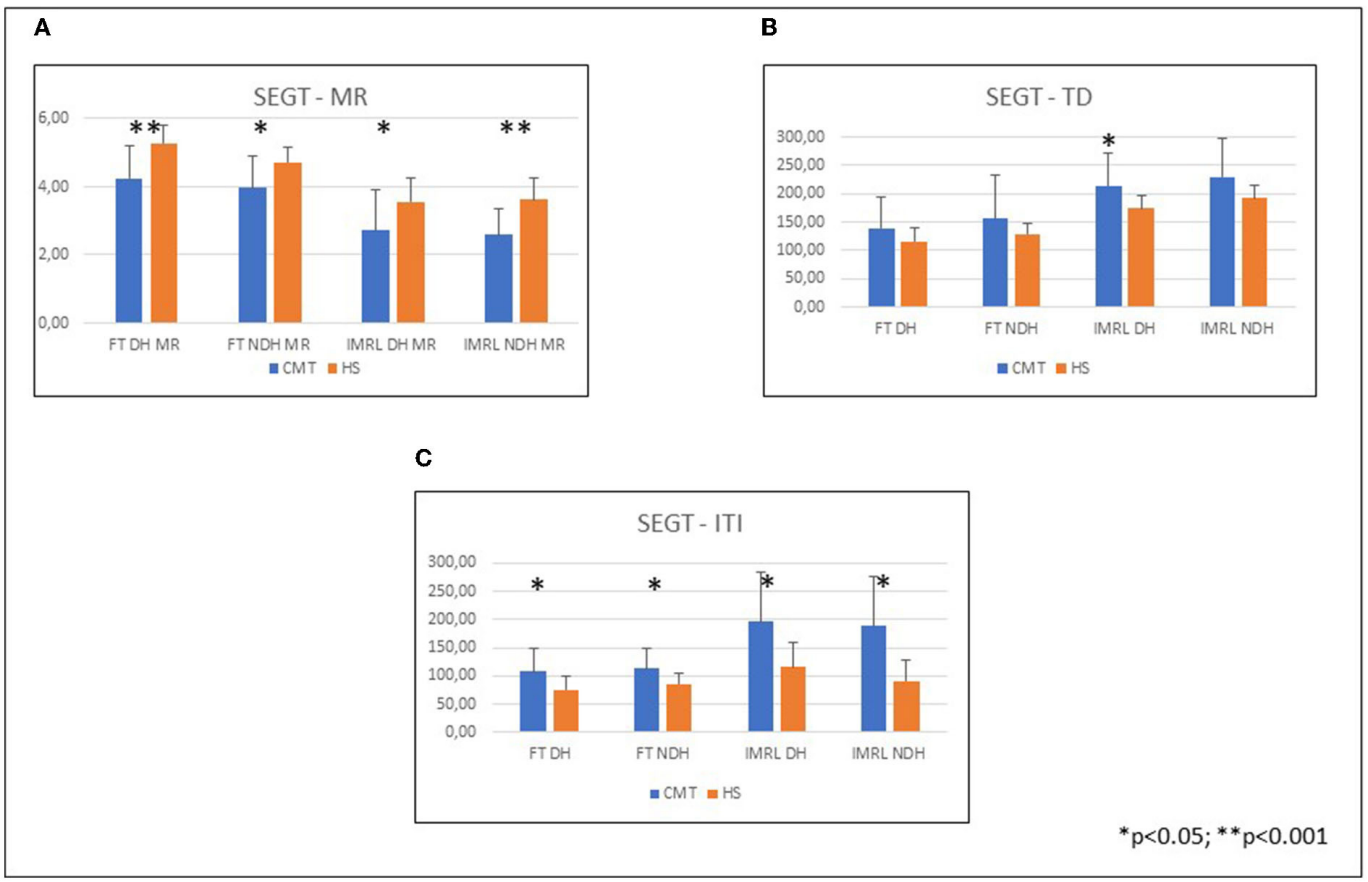
Hand performances at SEGT; CMT patients compared to healthy subjects. **(A)** Mean and SD of the SEGT-MR in the different groups: FT DH CMT 4.19 ± 1; HS 5.28 ± 0.56; FT NDH CMT 3.96 ± 0.94; HS 4.72 ± 0.47; IMRL DH CMT 2.66 ± 1.15; HS 3.57 ± 0.66; IMRL NDH CMT 2.56 ± 0.80; HS 3.62 ± 0.59. **(B)** Mean and SD of the SEGT-TD in the different groups: FT DH CMT 139.91 ± 53.07; HS 116.17 ± 23.91; FT NDH CMT 155.74 ± 72.71; HS 128.76 ± 22.12; IMRL DH CMT 216.48 ± 55.52; HS 173.99 ± 20.47; IMRL NDH CMT 230.44 ± 55.52; HS 192.14 ± 19.99. **(C)** Mean and SD of the SEGT-ITI in the different groups: FT DH CMT 107.79 ± 38.63; HS 75.72 ± 22.86; FT NDH CMT 112.33 ± 33.93; HS 85.32 ± 17.17; IMRL DH CMT 202.02 ± 89.47; HS 116.22 ± 41.37; IMRL NDH CMT 198.74 ± 114.88; HS 91.03 ± 31.98. CMT, Charcot-Marie-Tooth; HS, Healthy subjects; SEGT, sensor-engineered glove test; MR, movement rate; TD, touch duration; ITI, inter-tapping interval; DH, dominant hand; NDH, non dominant hand.

CMT patients showed worse performance than normal controls at the SEGT (Figure 2) in all sequences except TD (Table 2).

**Table 2:** CMT performances compared to HS in both clinical and instrumental OMs.

	**HS**	**CMT**	***p*-value**
TOT DH, mean (SD)	10 (0)	9.05 (1.43)	**0.019**
TOT NDH, mean (SD)	10 (0)	9.18 (1.18)	**0.005**
Trip Pinch DH, mean (SD)	96.25 (28.32)	76.77 (38.21)	0.132
Trip Pinch NDH, mean (SD)	86.57 (34.52)	70.52 (37.96)	0.233
Hand Grip DH, mean (SD)	102.10 (39.65)	91.52 (42.18)	0.481
Hand Grip NDH, mean (SD)	95.44 (39.50)	93.04 (39.02)	0.865
FT DH MR, mean (SD)	5.28 (0.56)	4.19 (1.00)	**0.001**
FT NDH MR, mean (SD)	4.72 (0.47)	3.96 (0.94)	**0.014**
IMRL DH MR, mean (SD)	3.57 (0.66)	2.66 (1.15)	**0.017**
IMRL NDH MR, mean (SD)	3.62 (0.59)	2.56 (0.80)	**0.000**
FT DH TD, mean (SD)	116.17 (23.91)	139.91 (53.07)	0.153
FT NDH TD, mean (SD)	128.76 (22.12)	155.74 (72.71)	0.222
IMRL DH TD, mean (SD)	173.99 (20.47)	216.48 (55.52)	**0.016**
IMRL NDH TD, mean (SD)	192.14 (19.99)	230.44 (66.29)	0.061
FT DH ITI, mean (SD)	75.72 (22.86)	107.79 (38.63)	**0.013**
FT NDH ITI, mean (SD)	85.32 (17.17)	112.33 (33.93)	**0.015**
IMRL DH ITI, mean (SD)	116.22 (41.37)	202.02 (89.47)	**0.004**
IMRL NDH ITI, mean (SD)	91.03 (31.98)	198.74 (114.88)	**0.003**

The statistically significant values are in bold.

### OMs and QoL

**Figure 3:**
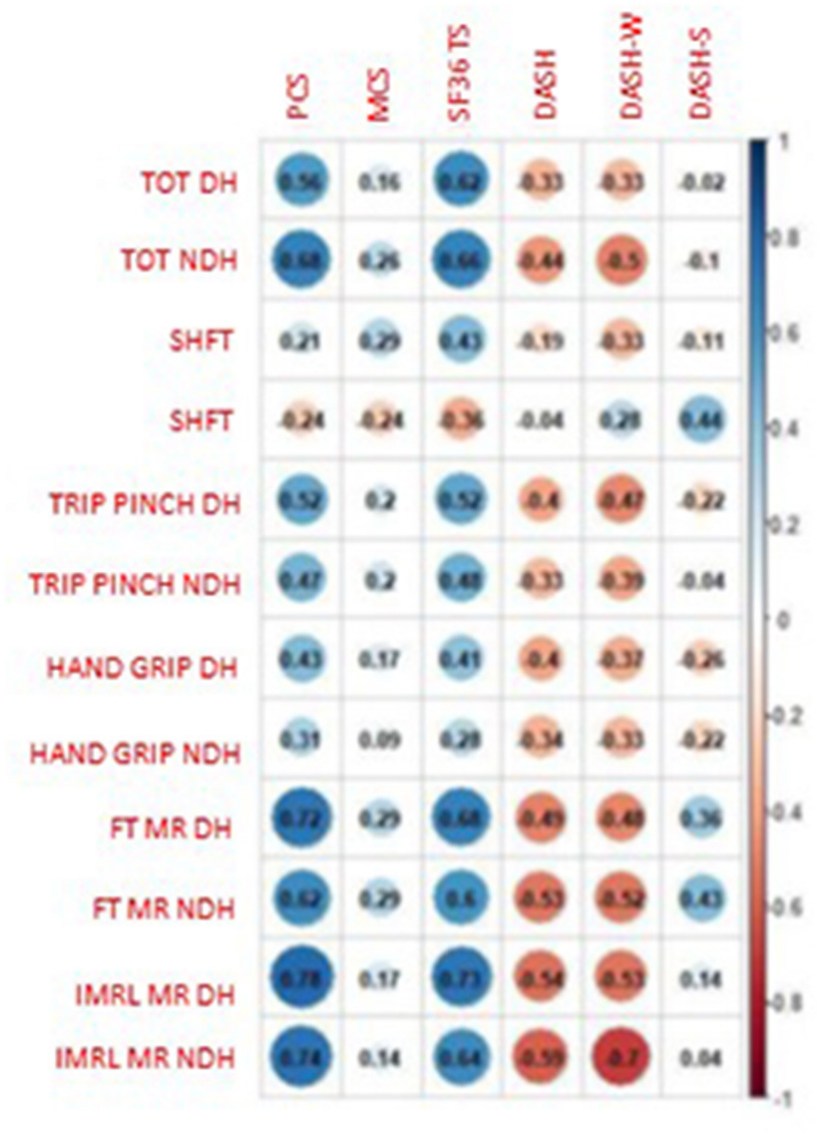
Correlations between clinical and instrumental Oms and Qol. PCS, physical composite score; MCS, mental composite score; TS, total score; DASH, Disability of Arm, Shoulder and Hand; DASH-W, DASH work; DASH-S, DASH sport; TOT, Thumb opposition test; SHFT, Sollerman hand function test; Trip pinch, tripod pinch; FTMR, finger tapping movement rate; IMRL MR, Index, medium, ring, little movement rate; DH, dominant hand; NDH, non-dominant hand.

All results are reported in Figure 3.

We found moderate correlations between SF36 total score and TOT both DH (rho: 0.62, *p* = 0.0027) and NDH (rho: 0.66, *p* = 0.0010) and low correlation with SHFT (rho: 0.43, *p* = 0.0515).

PCS presented moderate correlation with TOT both DH and NDH (TOT DH: rho: 0.56, *p* = 0.0077; NDH: rho: 0.68, *p* = 0.0007).

MCS presented only negligible correlations with other parameters.

DASH correlated with TOT and SHFT. Particularly, the TOT DH had low negative correlation with DASH (rho: −0.33, *p* = 0.1491) and DASH work (rho: −0.33, *p* = 0.1834), TOT NDH had low negative correlation with DASH (rho: −0.44, *p* = 0.0543) and moderate negative correlation with DASH work (rho: −0.50, *p* = 0.0349). DASH work had low negative correlation with SHFT (rho: −0.33, *p* = 0.1863).

### Dynamometer and QoL

All results are reported in Figure 3.

Tripod pinch DH had moderate correlation with PCS (rho: 0.52, *p* = 0.0165) and SF36 total score (rho: 0.52, *p* = 0.0146), negative low correlation with DASH (−0.40, *p* = 0.0847) and with DASH work (−0.47, *p* = 0.0489).

Tripod pinch NDH had low correlation with PCS (rho: 0.47, *p* = 0.0323) and SF36 total score (rho: 0.48, *p* = 0.0262), low negative with DASH (−0.33, *p* = 0.1596) and DASH work (−0.39, *p* = 0.1140).

Hand grip DH showed low correlation with PCS (rho: 0.43, *p* = 0.0497) and SF36 total score (rho: 0.41, *p* = 0.0669), negative low correlation with DASH (rho: −0.40, *p* = 0.0846) and DASH work (rho: −0.37, *p* = 0.1311).

Hand grip NDH showed low correlation with PCS (rho: 0.31, p=0.1698), low negative correlation with DASH (rho: −0.34, *p* = 0.1423) and Dash work (rho: −0.33, *p* = 0.1835).

### SEGT and QoL

All results are reported in Figure 3.

The SEGT both DH and NDH showed high correlation with PCS. Particularly, PCS had high correlation with FT DH (rho: 0.72, *p* = 0.0002) NDH (rho: 0.62, *p* = 0.0025), with IMRL DH (rho:0.78, *p* < 0.0001) and IMRL NDH (rho: 0.74, *p* = 0.0001). SEGT had high correlation also with SF36 total score (FT DH: rho: 0.68, *p* = 0.0007; FT NDH: rho: 0.60, *p* = 0.0043; IMRL DH: rho: 0.73, *p* = 0.0002; IMRL NDH: rho: 0.64, *p* = 0.0020). Negligible correlations between SEGT and MCS.

The FT MR DH had significant negative moderate correlation with DASH (rho: −0.49, *p* = 0.0278) and DASH work (rho: −0.48, *p* = 0.0438); in the NDH it had significant negative moderate correlation with DASH (rho: −0.53, *p* = 0.0163) and significant high negative correlation with DASH work (rho: −0.52, *p* = 0.0255).

The IMRL MR DH had significant negative moderate correlation with DASH (rho: −0.54, *p* = 0.0139) and DASH work (rho: −0.53, *p* = 0.0228). The NDH had significant negative moderate correlation with DASH (rho: −0.59, *p* = 0.0057) and significant high negative correlation with DASH work (rho: −0.70, *p* = 0.0013).

### DASH and SF-36

**Figure 4:**
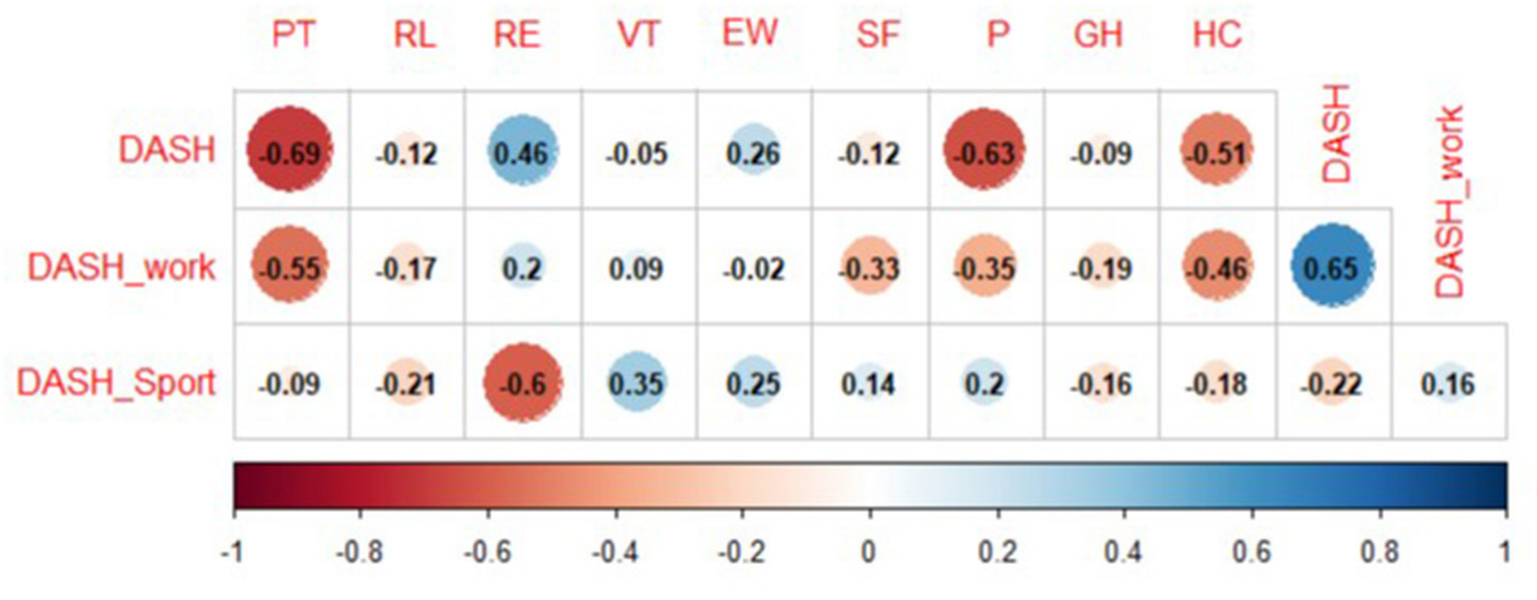
Correlations between subjective outcome measures. DASH, Disability of Arm, Shoulder and Hand; PF, physical functioning; RP, role-physical; RE, role-emotional; VT, vitality; EW, emotional well-being; SF, social functioning; P, pain; GH, general health; HC, health change.

All results are reported in Figure 4.

DASH showed significant negative moderate correlation with PF (rho: −0.69, *p* = 0.0007) and P (rho: −0.63, *p* = 0.0026), significant low correlation with RE (0.46, *p* = 0.0420) and significant negative moderate correlation with HC (rho: −0.51, *p* = 0.0229). DASH work showed significant negative moderate correlation with PF (rho: −0.55, *p* = 0.0183) and a moderate correlation with HC (rho: −0.46, *p* = 0.0520).

## Discussion

The hand is essential for the independence and quality of life. The hand grip function is a complex task requiring approaching, grasping, moving, and releasing phases (20).

The results of our work confirm what has been highlighted in previous studies (11, 12), that both clinical and instrumental tests are able to discriminate between healthy subjects and patients affected by CMT. In fact, as expected, in CMT patients all performances in clinical tests were significantly impaired compared to normal controls in which we did not find differences between DH and NDH and the average value was in the normal limits. At the dynamometric evaluation, in both hand grip and tripod pinch, CMT patients showed less strength than healthy controls; while in CMT patients the strength measured at the dynamometer were similar in DH and NDH, in healthy subjects DH was stronger than NDH. The SEGT, in addition to distinguish between healthy subjects and CMT, can also differentiate CMT patients with clinically unaffected and affected or mildly affected hands, as previously reported (11–13), and this may be particularly useful in order to minimize functional deficits with a tailored rehabilitative intervention (6, 9).

QoL is an important parameter to consider to better estimate the effectiveness of rehabilitation treatments and their impact on the patient. Generic health-related QoL OMs would not be suitable for clinical trials, as they lack specificity and sensitivity (21) to disease-related changes. There are currently no CMT-specific QoL scales available and therefore we chose SF36 which is the most used OM to evaluate the perception of the quality of life in clinical trials (22). Despite this, we found that total score and PCS of SF36 correlated with all clinical and instrumental OMs. In particular, we want to highlight the correlation with TOT, dynamometer and SEGT. Tripod pinch strength and thumb opposition are considered major determinants of manual dexterity in CMT and the correlation with SF36 suggests that this scale is therefore able to highlight the impact of upper limb disability in these subjects. We did find only a low correlation with hand grip, but this is not surprising since the Pinch Gauge dynamometer consider the intrinsic muscles of the hand, which are the earliest affected in the disease, while the extrinsic muscles are affected later (9, 23, 24). CMT subjects with significant intrinsic muscle damage lost lateral pinch strength but retained grip strength and that is due to the ability to compensate the intrinsic muscles deficit by increasing the activity of the extrinsic muscles. As for the SEGT, we found high correlations with the total score and PCS of SF36, thus confirming that the SEGT is a measure of dexterity able to discriminate upper limbs disability. Subjects with worse SEGT performances perceived a worse QoL, having greater motor impairment. We did not find correlation with the MCS and that may be explained because, as already stated, SF36 is a generic quality of life outcome measure and may be not enough sensitive to measure the variations or the impact of disability on the patient's emotional component.

DASH and SF36 relate to each other, but this is not surprising since hand dexterity is a fundamental requirement for autonomy in the ADL (5, 9, 11, 25). CMT persons who perceive limitations in manual skills report lower scores in QoL assessment, while subjects with better manual performances report less perception of limitation to DASH and SF36.

DASH scale correlates with all clinical and instrumental OMs except SHFT, and that lack of correlation may be due to the ceiling effect of SHFT which therefore fails to detect the variations at the highest levels of the function, limitation however perceived by the patient. An interesting result is that clinical and instrumental OMs also correlate with DASH work, but this is not surprising since CMT affects young patients engaged in work activities and it is therefore normal to find correlations between the deficit of fine dexterity and the ability of the upper limb during work. The fact that DASH sport has no correlation except with SEGT at FT MR, could be due to the very limited number of subjects (17%) practicing sport. It should be noted that the strongest correlations were found with FT MR and IMRL MR, confirming the SEGT as the most sensitive OM for upper limb assessment in these patients. However, we found a low correlation with the TOT and the dynamometer, even in DH. This could be due to the limited number of subjects recruited or else the subjects recruited may have had a low hand impairment. It is also possible, as already stated in previous studies (15), that persons with CMT have gradually adapted to a life influenced by their slowly progressive disease and therefore score lower than expected considering their poor performance on the hand function tests. Nevertheless, in literature most of the studies include patients affected by CMT1A, while in our study the numerically greater sub-population was composed of CMT1X, in which the distal impairment of the upper limb is more relevant and more frequent. This leads us to suggest that DASH may not be the best scale for remote monitoring of upper limb disorders in CMT patients.

The most important limitation of the present study is the small number of recruited subjects. Having a numerically greater sample could have allowed us to verify the differences between the different subgroups of patients.

The evaluation of the upper limb in the CMT is of primary importance. Often these patients present subclinical disorders or report difficulties in manipulating objects, with little evidence in the most used outcome measures. People with CMT often have physical limitations that can restrict their ability to travel to centers of excellence to perform periodical assessments and to undergo specifically tailored rehabilitation treatment. In addition, recent events such as the COVID-19 pandemic have made even more clear that there is an urgent need for trial OMs that do not require in-clinic visits and can be assessed remotely (26). Furthermore, an accurate assessment of the functionality of the hand could have important implications in designing a personalized rehabilitation treatment and assessing the possible improvement. An OM able to assess upper limb disability that can be administered remotely could have a major impact in monitoring patients with CMT. DASH scale may be used until a better and more specific and sensitive OM is found. However, the results of our study confirm the usefulness of SF36 in recognizing the impact of hand impairment in the QoL of CMT patients and lead us to suggest its use in the remote monitoring of physical functioning.

## Data availability statement

The raw data supporting the conclusions of this article will be made available by the authors, without undue reservation.

## Ethics statement

The studies involving human participants were reviewed and approved by CER Liguria. The patients/participants provided their written informed consent to participate in this study.

## Author contributions

LMo was responsible for research design, data analysis, and wrote the manuscript. CS contributed to the data interpretation. PM was responsible for statistical analysis. AA, ML, and FC performed all clinical and instrumental evaluations. SM and MG performed neurological evaluation and recruited the subjects. LMa and CT being expert neurologists and revised the manuscript. AS supervised the research. All authors contributed to the article and approved the submitted version.

## Funding

This work was supported by the Italian Ministry of Health, Bando Giovani Ricercatori 2013—Research Type: Clinical Health Care Research (Grant No. GR-2013-02357528).

## Conflict of interest

The authors declare that the research was conducted in the absence of any commercial or financial relationships that could be construed as a potential conflict of interest.

## Publisher's note

All claims expressed in this article are solely those of the authors and do not necessarily represent those of their affiliated organizations, or those of the publisher, the editors and the reviewers. Any product that may be evaluated in this article, or claim that may be made by its manufacturer, is not guaranteed or endorsed by the publisher.
